# MicroRNA-940 suppresses prostate cancer migration and invasion by regulating MIEN1

**DOI:** 10.1186/1476-4598-13-250

**Published:** 2014-11-19

**Authors:** Smrithi Rajendiran, Anil V Parwani, Richard J Hare, Subhamoy Dasgupta, Rhonda K Roby, Jamboor K Vishwanatha

**Affiliations:** From the Department of Molecular and Medical Genetics and Institute for Cancer Research, University of North Texas Health Science Center, 3500 Camp Bowie Blvd, Fort Worth, TX 76107 USA; From the Department of Pathology, UPMC Shadyside Hospital, Pittsburgh, PA 15232 USA; From the Department of Pathology, Plaza Medical Center, Fort Worth, TX 76104 USA; Department of Molecular and Cellular Biology, Baylor College of Medicine, Houston, TX 77030 USA; J. Craig Venter Institute, LaJolla, CA 92037 USA

**Keywords:** Migration, Invasion, Post-transcription regulation, Prostate cancer, MicroRNA, MIEN1, miRNA-940

## Abstract

**Background:**

MicroRNAs (miRNAs) are crucial molecules that regulate gene expression and hence pathways that are key to prostate cancer progression. These non-coding RNAs are highly deregulated in prostate cancer thus facilitating progression of the disease. Among the many genes that have gained importance in this disease, Migration and invasion enhancer 1 (MIEN1), a novel gene located next to HER2/*neu* in the 17q12 amplicon of the human chromosome, has been shown to enhance prostate cancer cell migration and invasion, two key processes in cancer progression. MIEN1 is differentially expressed between normal and cancer cells and tissues. Understanding the regulation of MIEN1 by microRNA may enable development of better targeting strategies.

**Methods:**

The miRNAs that could target MIEN1 were predicted by *in silico* algorithms and microarray analysis. The validation for miRNA expression was performed by qPCR and northern blotting in cells and by *in situ* hybridization in tissues. MIEN1 and levels of other molecules upon miRNA regulation was determined by Western blotting, qPCR, and immunofluorescence. The functional effects of miRNA on cells were determined by wound healing cell migration, Boyden chamber cell invasion, clonal and colony formation assays. For knockdown or overexpression of the miRNA or overexpression of MIEN1 3′UTR, cells were transfected with the oligomiRs and plasmids, respectively.

**Results:**

A novel miRNA, hsa-miR-940 (miR-940), identified and validated to be highly expressed in immortalized normal cells compared to cancer cells, is a regulator of MIEN1. Analysis of human prostate tumors and their matched normal tissues confirmed that miR-940 is highly expressed in the normal tissues compared to its low to negligible expression in the tumors. While MIEN1 is a direct target of miR-940, miR-940 alters MIEN1 RNA, in a quantity as well as cell dependent context, along with altering its downstream effectors. The miR-940 inhibited migratory and invasive potential of cells, attenuated their anchorage-independent growth ability, and increased E-cadherin expression, implicating its role in mesenchymal-to-epithelial transition (MET).

**Conclusions:**

These results, for the first time, implicate miR-940, a regulator of MIEN1, as a promising novel diagnostic and prognostic tool for prostate cancer.

**Electronic supplementary material:**

The online version of this article (doi:10.1186/1476-4598-13-250) contains supplementary material, which is available to authorized users.

## Background

Metastatic progression of prostate cancer is a major cause of death among men in the United States [1]. Though cancer metastasis is a highly complex multi-step process facilitated by several key events and molecular players, the most effective way known to prevent this progression is by identifying and targeting the various genes involved in the process(es) [2, 3]. Gene regulation is tightly controlled in the normal cells, thereby retaining the homeostatic expression of the appropriate genes for the functioning of the organism. Deregulation of these mechanisms in cancer causes the disrupted expression of the genes, which in turn furthers the cancer progression. MicroRNAs are a class of endogenous, small non-coding RNAs, 18 to 22 nucleotides long in their mature form, which can regulate a set of target genes and result in translational repression or mRNA degradation depending on the extent of complementarity and cellular context [4, 5]. Recent studies have shown extensive dysregulation of miRNAs in prostate cancer [6–8]. Many miRNAs have been implicated as tumor suppressors or oncomiRs depending on their target(s) and/or the global effects they have towards cancer progression [9–12]. Though studies have been performed with respect to certain miRNAs and their specific targets in prostate cancer [13, 14], not much is known about novel miRNAs targeting the players of cancer progression that can be used as diagnostic markers for early detection, or detection of a possible recurrence or resistance, or therapeutic agents to slow the progression. Identification of these novel miRNAs and their target gene(s), and the pathways they affect during cancer progression, will provide new insights into using them for diagnosis or determination of specific therapy regimens.

Migration and invasion enhancer 1 (*MIEN1*), alternately called C17orf37, C35, RDX12, XPT4, ORB3 or MGC14832, is located in the 17q12-21 region of the human chromosome next to HER2/*neu* in a tail-to-tail arrangement. MIEN1 is abundantly expressed in different stages and grades of prostate cancer phenotypes when compared to normal cells and tissues [15]. MIEN1 has also been predicted as a novel breast cancer biomarker with increased expression in patients with metastatic progression to lung and liver, suggesting its importance in cancer metastasis [16]. MIEN1 plays a role in prostate cancer migration and invasion through enhancement of filopodia formation by facilitating actin cytoskeletal rearrangement and by up-regulating the Akt dependent NF-κB target genes [15, 17]. This was further confirmed by the recent determination of the solution structure of MIEN1 which predicts that Akt phosphorylation via MIEN1 may be dependent on the active redox-like motif in the MIEN1 structure [18]. MIEN1 is also post-translationally modified by prenylation, via GGTase-I, at its C-terminus CVIL motif. Deletion of the motif not only led to the disruption of MIEN1 membrane localization and reduced invasive and migratory potential but also decreased metastasis to the lungs [17]. Although abrogation of prenylation is a possible targeting strategy, it cannot be effectively used since it has been proven that many proteins involved in the regular functioning of the cell are prenylated, rendering this a very important modification. Hence, inhibition of prenylation could negatively impact multiple cellular processes [19]. On the contrary, since MIEN1 is differentially expressed between normal and cancer cells and tissues, deciphering the regulatory mechanism(s) that explain the aberrant expression of MIEN1 in cancer will enable targeting MIEN1 using mechanisms that are endogenously prevalent thus forming an intervention for prostate cancer progression.

In this study, we have identified a novel miRNA, hsa-miR-940 (miR-940), which targets and regulates MIEN1 expression. Our study indicates that miR-940 expression inversely correlates with tumor progression in clinical prostate cancer and the loss of miR-940 in cancer causes an increased expression of MIEN1 which in turn enables prostate cancer progression. Ectopic expression of miR-940 resulted in not only decreased MIEN1 and its downstream effector molecules, but also reduced the migratory and invasive potential of the cells. Though the overall proliferation was unaltered, the ectopic expression of miR-940 reduced the anchorage-independent growth of cells, increased E-cadherin and decreased slug expression, suggesting facilitation of mesenchymal-to-epithelial transition (MET). Our results demonstrate that miR-940 may be a useful diagnostic marker as well as a therapeutic agent for prostate cancer.

## Results

### MIEN1 is post-transcriptionally regulated by microRNAs

**Figure 1:**
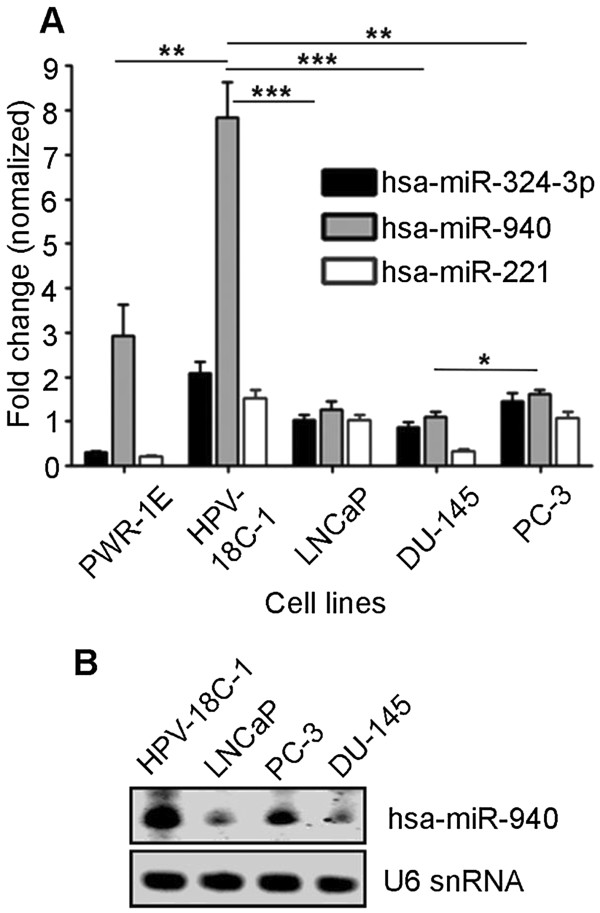
**miR-940 expression** ***.*** **(A)** qPCR shows expression of the three identified microRNAs: hsa-miR-324-3p, hsa-miR-940 and hsa-miR-221 normalized to U6 snRNA in immortalized prostate derived cell lines, PWR-1E and HPV-18C-1 and different prostate cancer cells, LNCaP, DU-145 and PC-3. **(B)** Northern Blot depicting the expression of hsa-miR-940 in immortalized prostate cell line HPV-18C-1 and prostate cancer cells LNCaP, PC-3 and DU-145. ***P ≤0.001; **P ≤0.01; *P ≤0.05.

In various androgen dependent and castration-resistant prostate cancer cells, both MIEN1 mRNA and protein are highly expressed compared to the immortalized normal cells of the prostate [15]. Interestingly, in PC-3 cells, which are androgen receptor negative, though MIEN1 mRNA was expressed, the protein was absent. Hence, we predicted an active role of post-transcriptional regulation of MIEN1. Downregulation of microRNA processing restriction endonucleases, Drosha and/or Dicer [5] using RNAi resulted in a significant transcriptional up-regulation of MIEN1 in HEK-293T cells, which do not express MIEN1 mRNA or protein (Additional file 1: Figure S1A). In PC-3 cells, the knockdown of the miRNA maturation enzymes resulted in an increase in MIEN1 protein expression by ~4-fold (Additional file 1: Figure S1B). We next performed a microarray analysis to determine the miRNAs that were differentially expressed between immortalized normal cells (PWR-1E) and cancer cells (DU-145). The raw data of the miRNA expression analysis is available at the NCBI’s Gene Expression Omnibus with GEO accession number GSE62286. Subsequently, using BLAST and *in silico* algorithm-based predictions, we identified three microRNAs, hsa-miR-324-3p, hsa-miR-221, and hsa-miR-940, that were differentially expressed between DU-145 and PWR-1E cells and could potentially target MIEN1 3′UTR (Additional file 2: Figure S2A and S2B) [20, 21]. Using qPCR, the expression levels of these microRNAs were quantitated as fold change normalized to U6 snRNA in the different cell lines. A significantly higher expression of miR-940 was observed in the non-malignant cells, PWR-1E (~3-fold) and HPV-18C-1 (~7.5-fold), compared to DU-145 and LNCaP, while, PC-3 showed ~1.5-fold higher expression of miR-940 (Figure 1A). The expression of miR-221 and miR-324-3p were neither consistently higher in the immortalized cells compared to the cancer cells, nor were they significantly different, together indicating that miR-940 may be the most relevant regulator of MIEN1 among the three miRNAs. Next, we performed northern blotting to confirm the expression levels of the 21nt miR-940 with a biotin-labeled probe. Consistent with the pattern observed by PCR, the expression of miR-940 in HPV-18C-1 was significantly higher (Figure 1B).

**Figure 2:**
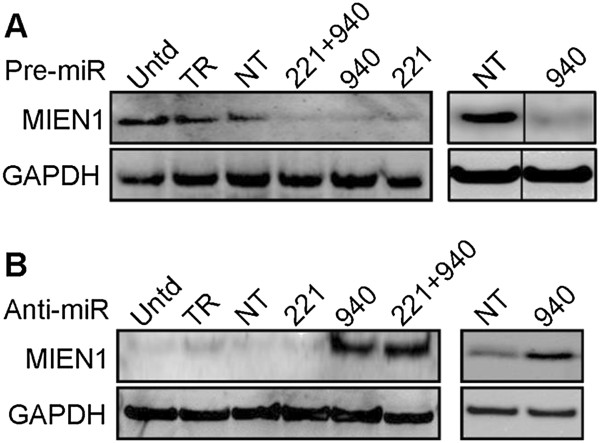
**MIEN1 expression upon transfection of miRNA mimic(s) or inhibitor(s) in different cell lines** ***.*** **(A-B)** Western blot reveals levels of MIEN1 in the cell lines transfected with miR-221 (221), miR-940 (940) or the combination (221 + 940) while untransfected (Untd), scrambled miR (NT) or transfection reagent (TR) transfected cells are controls in **(A)** DU-145 (**left**), LNCaP (**right**) and **(B)** PC-3 (**left**), PWR-1E (**right**). GAPDH was used for normalization.

To validate the regulation of MIEN1 by miRNA(s), we ectopically overexpressed the miRNA mimics or inhibitors in the various cell lines. We observed a decrease in the expression of MIEN1 protein by ~3- and ~2-fold in DU-145 (Figure 2A, left) and LNCaP (Figure 2A, right) cells, respectively, when transfected with miR-940 mimic. Conversely, inhibiting the endogenous miR-940 in PC-3(Figure 2B, left) and PWR-1E (Figure 2B, right) using anti-miR-940 increased the MIEN1 protein by ~4- and ~2-fold, respectively. Since miR-221, was significantly higher in PC-3 compared to DU-145, we also ectopically expressed miR-221 mimic in DU-145, together with miR-940, or by itself, and observed a decrease in the MIEN1 protein (Figure 2A, left). But, when we inhibited miR-221 alone in PC-3, we did not see any increase in MIEN1 (Figure 2B, left), implying miR-940 to be a more potent regulator of MIEN1 than miR-221. Together, these results demonstrate that miR-940 is differentially expressed between normal and cancer cells and that it targets and regulates MIEN1 expression. Hence, from here on, we only examined the relevance and role of miR-940 in key processes of prostate cancer progression.

### Loss of microRNA, hsa-miR-940, is an indicator of prostate cancer

**Figure 3:**
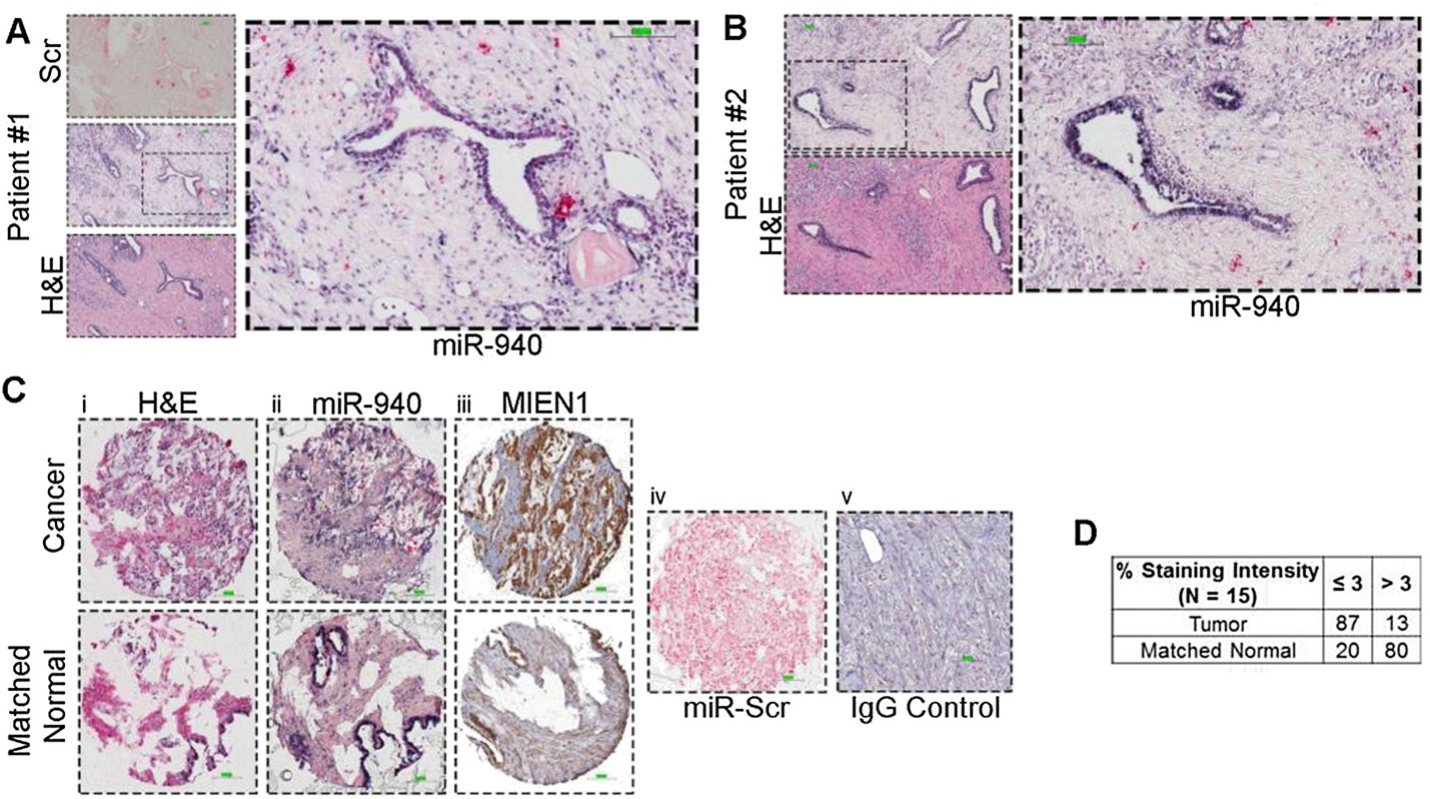
**miR-940 expression in human prostate cancer and normal tissues** ***.*** **(A-B)** *In situ* hybridization of the scrambled miR (miR-Scr) or miR-940 and H&E staining in normal/benign glands compared to the infiltrating tumor. **(C)** H&E staining (**i**), miR-940 (**ii**), and MIEN1 (**iii**) in cancer (**top**) and matched normal (**bottom**) tissues with miR-Scr (**iv**), and IgG control (**v**) representing the negative controls. **(D)** ~87% (n =13) tumor shows low (intensity ≤3) expression of miR-940 compared to ~80% (n =12) of the matched normal tissue with higher (intensity >3) miR-940 expression; conversely, ~13% (n =2) of the tumor expressed more (>3) miR-940 compared to lower expression (≤3) in ~20% (n =3) of the matched normal (n =15). Scale bar: 100μm.

The expression levels of miR-940 were next examined in a clinical sample cohort of prostate cancer patient tissues by *in situ* hybridization with miR-940 or scrambled miRNA probe as described in the methods. The prostate cancer (PCa) and matched normal (MN) tissues were independently scored and graded (based on the corresponding H&E performed) by two pathologists; only those that matched were used to draw conclusions. In two specific patients who had undergone surgical resection via radical prostatectomy, we observed that miR-940 expression was high in normal glands and benign prostatic hyperplasia with the expression being lower in infiltrating prostate cancer cells (Figure 3A and B). In the small pilot cohort of 15 samples, we observed that the miR-940 expression was higher in the matched normal sections in contrast to the low expression in the tumor cells (represented in Figure 3C). Correspondingly, the expression of MIEN1 was relatively higher in the cancer sections compared to the normal (represented in Figure 3C). We observed that the miR-940 staining intensity was high (4 and 5) in 12 of the normal tissues, while only 2 out of 15 tumor sections showed staining intensity of 4. Complementarily, 3 of the 15 normal tissues showed a staining intensity of 3 as opposed to tumors exhibiting lower intensities (1, 2, and 3) in 13 of the cases (Figure 3D). Additionally, to determine if miR-940 levels vary in benign prostatic hyperplasia (BPH) and metastatic prostate tumors (Mets), we performed *in situ* hybridization and immunohistochemistry and quantified the staining intensities of miR-940 as well as MIEN1 in these tissues. The representative images of the staining pattern are provided in Additional file 3: Figure S3A. As expected, in the small cohort of samples, a statistically significant difference was observed in the expression levels of both miR-940 as well as MIEN1 between the various groups. While there were higher levels of miR-940 in the BPH and MN tissues, with a reduction in PCa, the levels were minimal in Mets (Additional file 3: Figure S3B,i). Conversely, the highest expression of MIEN1 was observed in the metastatic tissues (Mets) followed by PCa tissues, with lowest expression in the BPH and MN tissues (Additional file 3: Figure S3B,ii). Together, our results indicate that even in a clinical setting (supported by the *in vitro* data), miR-940 expression is consistently higher in the normal tissues as opposed to the tumor cells.

### MIEN1 is a direct target of miR-940

**Figure 4:**
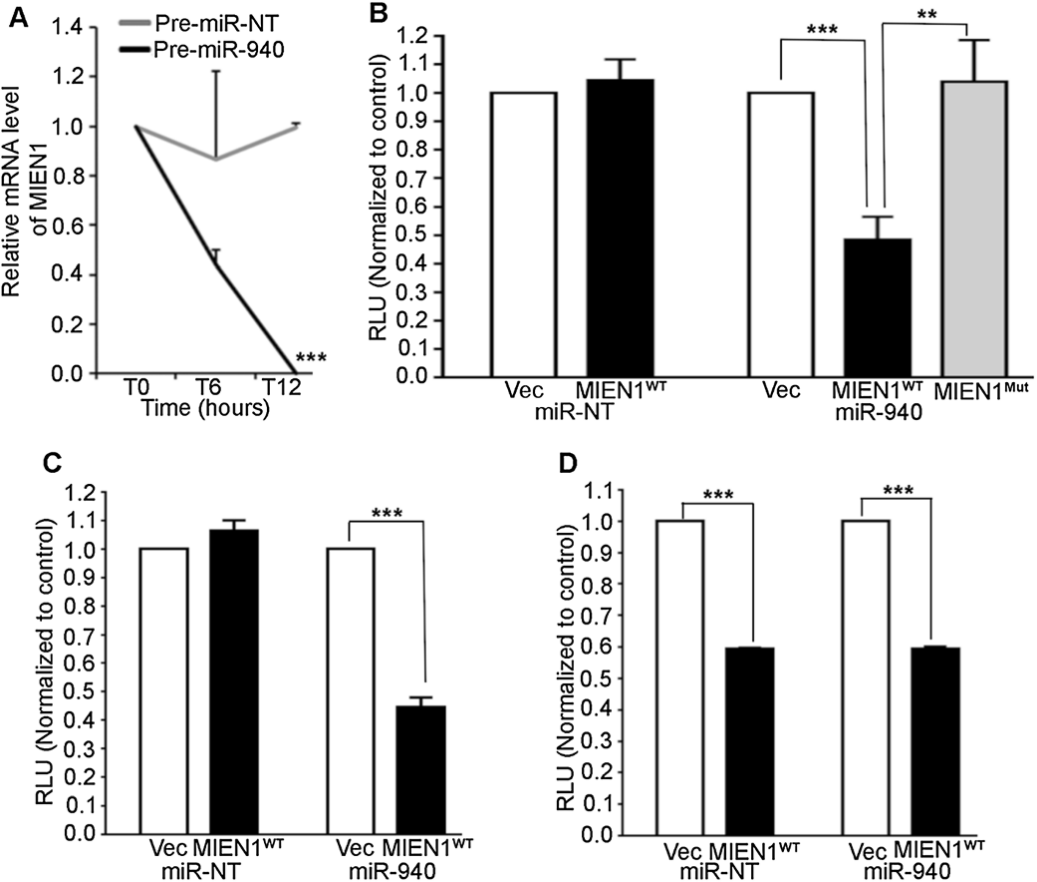
**miR-940 directly binds to MIEN1** ***.*** **(A)** MIEN1 mRNA level expressed as fraction of the initial value (T0) plotted over time, upon 10 μg/ml Actinomycin-D treatment following transfection with the Pre-miR-NT or Pre-miR-940 for 48 hours in DU-145. (**B-D**) Luciferase Reporter Assay showing relative luminescence upon co-transfection of miR-940 (**right**) or Pre-miR-NT (**left**) with either empty luciferase vector (Vec) or MIEN1 3′UTR luciferase vector (MIEN1^WT^ / MIEN1^Mut^) in DU-145 **(B)**, LNCaP **(C)** or PC-3 **(D)** respectively. ***P ≤0.001; **P ≤0.01.

Next, we wanted to determine if the MIEN1 mRNA stability was altered directly by miR-940. DU-145 cells were transfected with either miR-940 or the control miRNA and treated with Actinomycin-D (Act-D). The half-life of MIEN1 mRNA was observed to be ~6 hours with almost no detectable mRNA in 12 hours after treatment with Act-D in miR-940 transfected cells compared to the control transfected cells (Figure 4A), thus indicating that miR-940 decreases MIEN1 mRNA levels significantly.

We then examined if miR-940 directly binds to the MIEN1 3′UTR using a luciferase plasmid cloned with MIEN1 3′UTR. Binding of the miRNA directly to the 3′UTR of MIEN1 is expected to inhibit the luciferase luminescence compared to the luminescence when the miRNA is unable to bind to the empty luciferase vector control or the MIEN1 3′UTR containing a mutation in the binding site for miR-940. When MIEN1 3′UTR containing luciferase plasmid (MIEN1^WT^) was co-transfected with miR-940 in DU-145 (Figure 4B) cells, there was ~2-fold reduction in luminescence as opposed to luminescence from empty luciferase plasmid (Vec) or mutant MIEN1 3′UTR (MIEN1^Mut^) and miR-940 co-transfections. Additionally, co-transfection of the miR-NT with either the Vec or the MIEN1^WT^ plasmid did not show any significant changes in the luminescence in DU-145. Similarly, in LNCaP cells, miR-940 co-transfection with MIEN1^WT^ plasmid showed a reduction in luminescence compared to the controls (Figure 4C). In PC-3 cells, which express some endogenous miR-940, co-transfection of MIEN1^WT^ plasmid with either the miR-940 mimic or the control miRNA showed significantly lesser luminescence (Figure 4D) compared to the Vec plasmid co-transfections. Since we observed that the ectopic overexpression of miR-940 did not have a higher reduction in luminescence compared to the miR-NT co-transfections, it is possible that the regulation of MIEN1 in PC-3 cells may not necessarily involve mRNA degradation; it may just be translational repression. This conforms to our earlier findings that though the MIEN1 mRNA is exhibited in PC-3, protein is very low to negligible. Together, the luciferase assays provide direct evidence of miR-940 – MIEN1 mRNA interaction.

### miR-940 affects target genes in a cellular context dependent manner

**Figure 5:**
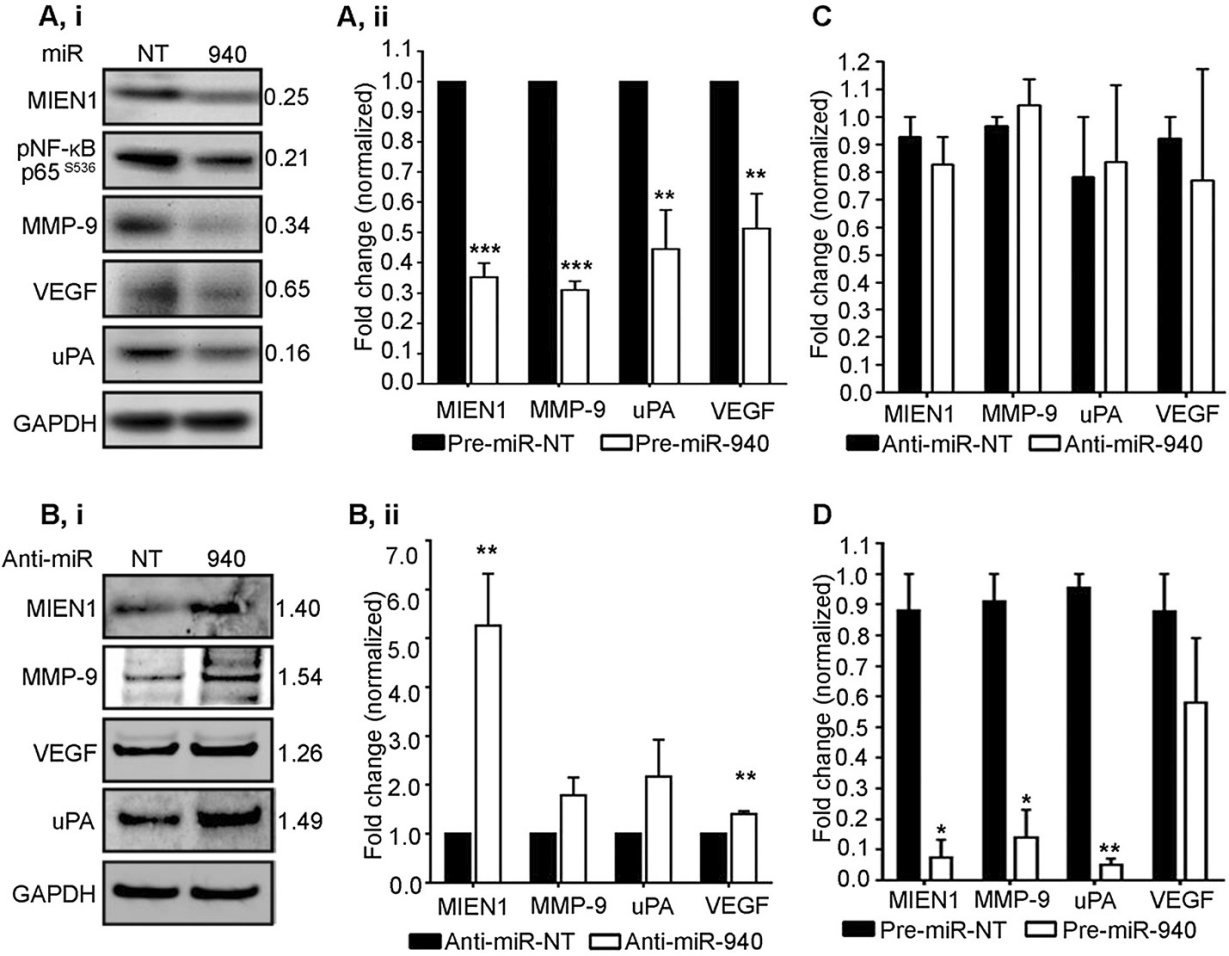
**miR-940 targets MIEN1 and affects MMP-9, uPA and VEGF expression in a cellular context-dependent manner** ***.*** (**A-B**) Expression of the downstream targets of MIEN1 upon transfection of Pre-miR-NT or Pre-miR-940 in DU-145 **(A)** and Anti-miR-NT or Anti-miR-940 in PWR-1E **(B)** at both the translational and transcriptional levels as shown by western blotting (**A,i, B,i**) and qPCR (**A,ii, B,ii**) respectively. **(C-D)** qPCR showing the expression of MIEN1 and the effectors upon transfection with Anti-miR-940 in PC-3 **(C)** and with Pre-miR-940 in PC-3 (**D**).***P ≤0.001; **P ≤0.01; *P ≤0.05.

Since it is known that silencing MIEN1 decreases NF-κB mediated downstream effectors MMP-9, uPA and VEGF [15], we examined if ectopic overexpression of miR-940 had the same effect on downstream effectors of MIEN1. Upon overexpression of miR-940 in DU-145, a decrease was observed in MMP-9, uPA and VEGF, along with pNF-κB S536 (an indicator of nuclear NF-κB that is responsible for the transcription of target genes) at the protein level (Figure 5A,i) compared to the control. Reductions from ~2- to 3-fold in MMP-9, uPA and VEGF transcripts in DU-145 (Figure 5A,ii) further confirmed the inhibition of the NF-κB mediated transcriptional activity. Conversely, the knockdown of endogenous miR-940 in PWR-1E increased protein (Figure 5B,i) and transcript levels (Figure 5B,ii) of MMP-9, uPA and VEGF.

Interestingly, the effects observed with PC-3 were quite unique. It is well known that the miRNA regulation can result in either mRNA degradation or translational repression of the target [22, 23]. Though PC-3 has an increased level of MIEN1 protein upon treatment with anti-miR-940 (Figure 2B, left), there was no increase in the mRNA levels of MIEN1 (Figure 5C). Further, ectopic overexpression of miR-940 in PC-3 resulted in a decrease in MIEN1 and its downstream targets, MMP-9, uPA and VEGF compared to the control at the mRNA level (Figure 5D), indicating that the mechanism by which miR-940 affects MIEN1 mRNA is dependent on the amount of miRNA present, which in turn is dependent on the cell type being considered and thus the general cellular context.

### miR-940 attenuates the migration and invasion of prostate cancer cells along with inhibiting their anchorage-independent growth potential

**Figure 6:**
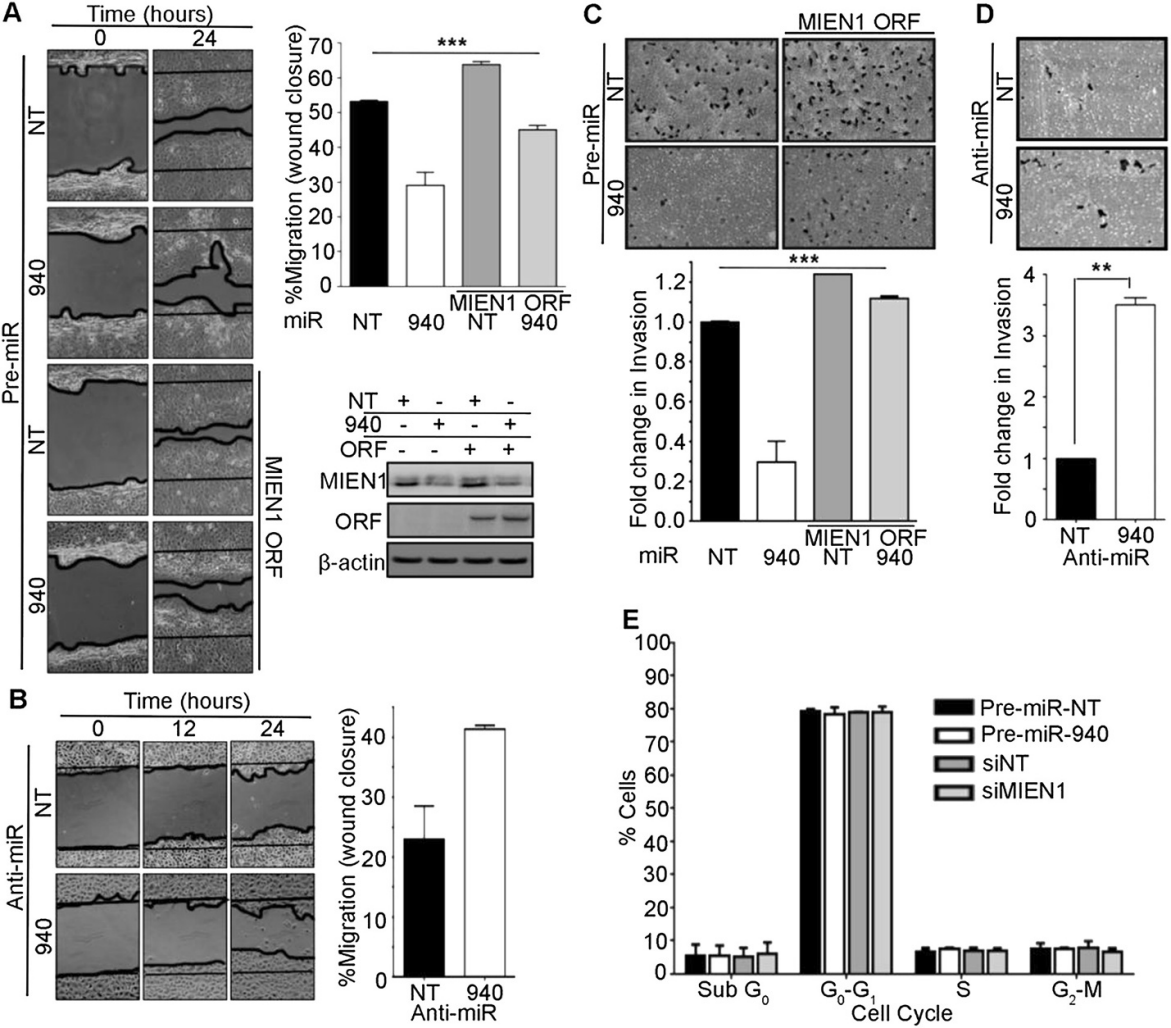
**miR-940 affects the cellular migratory and invasive potential** ***.*** **(A)** Migration of DU-145 upon transfection with either Pre-miR-NT, Pre-miR-940 or MIEN1 ORF with miR-NT or miR-940 depicted by a wound healing assay (**left**) and quantified as a percentage of the wound area closed (**right, top**) after 24 hours; (**right, bottom**) Western blotting to show MIEN1 expression in the different cells. **(B)** Representation (**left**) and quantification (**right**) of the migratory potential of PC-3 cells upon transfection with Anti-miR-NT or Anti-miR-940, 24 hours after making a wound. **(C-D)** Representation (**top**) and quantification (**bottom**) of the invasive potential of DU-145 **(C)** and PC-3 **(D)** cells as determined by the Boyden chamber matrigel invasion assay when transfected by miRNA mimic and inhibitor respectively. **(E)** Cell cycle analysis showing the percentage of the cell population in the Pre-miR-NT, Pre-miR-940, siNT and siMIEN1 transfected DU-145 cells. ***P ≤0.001; **P ≤0.01.

MicroRNAs have multiple targets; hence, the overall effects of a particular microRNA on global cellular functions may vary depending on the regulation of the various targets and their combined implications [24, 25]. Migration and invasion are key processes that facilitate cancer progression and MIEN1 is known to increase these processes [2, 15]. Since, MIEN1 is one of the direct targets of miR-940, we sought to determine if the ectopic overexpression of miR-940 could attenuate these processes, independent of the effect that miR-940 may have on other transcripts. A scratch wound healing migration assay showed that lesser migration (~0.54-fold) was observed in DU-145 cells treated with miR-940 compared to the non-targeting control 24 hours after the initial scratch (Figure 6A) while this effect was rescued partially (~0.86-fold) when the cells were transfected with MIEN1 ORF plasmid which was non-targetable by miR-940 since it lacks the 3′UTR. Knockdown of the miR-940 using anti-miR-940 in PC-3 resulted in ~1.8-fold increase in its migratory potential (Figure 6B). To investigate whether miR-940 affects the cell viability, MTT assays were performed with ectopic expression of miR-940 mimic in DU-145 and anti-miR-940 in PC-3 cells. Also, in DU-145 these results on cell proliferation were compared with knockdown of MIEN1 directly with siRNA. Though transfection with anti-miR-940 in PC-3 cells showed a statistically significant decrease in cell viability after 48 hours (Additional file 4: Figure S4A) this was only a 15% change and the effect was abrogated after 72 hours. No significant differences were observed in the cell viability after 48 or 72 hours of transfection in DU-145 (Additional file 4: Figure S4B), proving that neither miR-940, nor its target MIEN1, has any dramatic effect on cell viability. Next, the invasive potential of the cells was determined using miR-940 mimic or inhibitor transfected DU-145 or PC-3 cells through the transwell matrigel invasion assay system. The ectopic expression of miR-940 decreased the invasiveness of DU-145 -5-fold (Figure 6C), which was abridged by reintroduction of non-targetable MIEN1 ORF construct and conversely, the invasiveness of PC-3 was ~3.5-fold higher upon inhibition of endogenous miR-940 by anti-miR-940 (Figure 6D). Furthermore, to ensure that the effects observed on migration and invasion are a true result of these processes, cell cycle analysis was performed with DU-145 cells transfected with miR-940 and siRNA against MIEN1. Our results revealed no significant differences between the different populations of DU-145 cells (Figure 6E). Taken together, these results show that miR-940 inhibits both the migratory and invasive potential of the cells without affecting cell viability and that these responses are, at least partially, mediated through its regulation of MIEN1.

**Figure 7:**
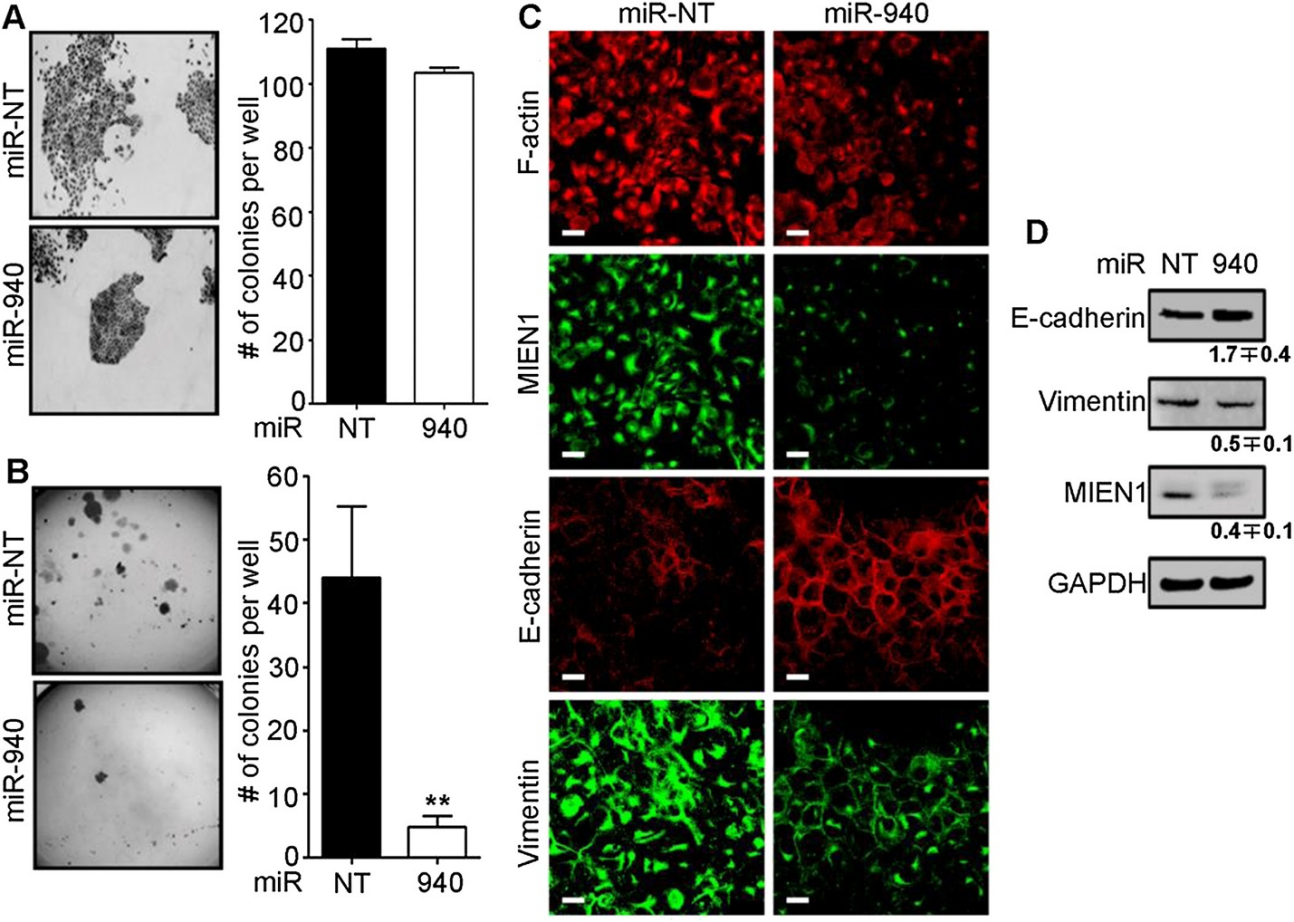
**miR-940 alters the anchorage-dependent and -independent growth of DU-145 cells** ***.*** **(A)** Morphology (**left**) and number (**right**) of colonies formed by Pre-miR-NT or Pre-miR-940 transfected DU-145 cells after 12 days on tissue culture treated adherent plates. **(B)** Soft agar colony formation assay shows the colonies in the agar (**left**) and their quantification (**right**) after 12 days. **(C-D)** Immunofluorescence **(C)** and western blotting **(D)** show the expression and localization of various proteins upon overexpression of Pre-miR-940 compared to Pre-miR-NT. Scale bar: 20μm. **P ≤0.01.

The ability of the cancer cells to adhere and grow by anchorage-dependent and -independent mechanisms is very important to determine their clonogenic ability and hence their potency to evade cell death and finally metastasize [26]. We observed that the DU-145 cells transfected with miR-940 formed smaller, smooth edged colonies compared to bigger, disseminated colonies formed by the control miR transfected cells, after 12 days (Figure 7A, left and Additional file 5: Figure S5A) under anchorage-dependent conditions. The total number of individual colonies remained unchanged between the treatments (Figure 7A, right). Conversely, the soft agar colony formation assay demonstrated that the anchorage-independent growth potential of the miR-940 transfected cells was highly hindered (~8-fold) compared to the control transfected cells after 12 days (Figure 7B). Dissemination of the cells is an indicator of the cells undergoing epithelial-to-mesenchymal transition (EMT), a phenomenon crucial to the initial detachment of the cells from the tumor site, leading to the invasion and migration of cells, and finally resulting in progression of cancer [27]. The considerable morphological difference observed in terms of compactness of the colonies between cells transfected with miR-940 and miR-NT suggested loss of the ability of the miR-940 transfected cells to undergo EMT. To further confirm the possibility of the involvement of miR-940 in hindering EMT, we performed immunostaining for E-cadherin, a cell adhesion marker that is downregulated if the cells undergo the process of EMT, and Vimentin, a mesenchymal marker. Our results show an increase in E-cadherin in miR-940 transfected cells compared to control cells, and a decrease in Vimentin along with disruption of its membrane localization (Figure 7C and D). Additionally, the mRNA expression of Slug, a transcriptional regulator of E-cadherin, decreased in miR-940 transfected cells compared to the control (Additional file 5: Figure S5B). In PC-3 cells, transfection of the miR inhibitor caused a decrease in E-cadherin transcript levels (Additional file 5: Figure S5C). Together, this suggests the possible involvement of miR-940 in MET, the reverse process of EMT.

## Discussion

MIEN1, a novel gene in the 17q12 region of the human chromosome, is differentially expressed between cancer and normal cells and tissues [16]. Previous studies indicate that MIEN1 plays an important role in prostate cancer progression [15, 17]. In this study, we show that MIEN1 undergoes post–transcriptional regulation by miR-940. Our data show that miR-940 decreases the migratory and invasive potential of prostate cancer cells along with facilitation of MET. Also, decreased expression of miR-940 in prostate cancer specimens proves the clinical relevance of this miRNA, leading to our belief that miR-940 is a potential diagnostic marker and therapeutic agent.

The use of miRNAs for therapy and/or diagnosis of cancer is currently under consideration due to the accumulating evidence demonstrating their extensive deregulation in many cancers, including prostate cancer [7, 28–30]. MiRNA mediated regulation of a target gene depends on multiple parameters including: 1) properties of miRNA responsive elements, such as the degree of complementarity and accessibility; 2) number of miRNAs that could target a single transcript; 3) expression of competitive endogenous mRNAs for a miRNA in a specific cellular context; 4) stimulus for the miRNA transcription/splicing and hence expression and stability; and, 5) other factors influencing the target mRNA stability and expression [22–25]. Careful examination of the degree to which a gene is regulated by a miRNA and the overall effects of the miRNA mimic or inhibitor, are essential to determine the global role of the miRNA in any cellular context.

The distinct difference in the expression of MIEN1 and lack of protein despite mRNA expression in PC-3 cells directly implied involvement of post-transcriptional regulation. Our experiments confirmed that MIEN1 is indeed regulated by miRNA and led to the identification and validation of miR-940. Alterations in mRNA half-life in the presence of the miRNA elucidated the effect of miR-940 on MIEN1 mRNA stability. Furthermore, using luciferase assays, we ascertained that miR-940 binds directly to the 3′UTR of MIEN1 to cause its suppression.

MIEN1 is minimally expressed in several normal tissues compared to its overexpression in cancer [16]. Its proximity to the HER2/*neu* locus on chromosome 17 explains its frequent amplification (in 79% of breast cancers) with HER2 amplicon [31]. A recent study using a set of eight genes, including MIEN1, revealed a moderate response to adjuvant Trastuzumab therapy even in HER2 negative breast cancer, confirming the importance of this gene in responses to neo-adjuvant therapies [32]. Katz *et al.* have shown that the overall survival of breast cancer patients is low in cases where MIEN1 is highly expressed while lower expression indicates better prognosis [33]. It is well known that prostate cancer-related deaths are due to metastasis rather than the presence of a primary tumor alone. Metastasis is a complex process involving multiple intermediate steps, from detachment of the cells at the primary site to formation of secondary tumor. The tumor cells evade the resistances faced at every step by different mechanisms [2, 3]. Our previous study has shown that cells overexpressing MIEN1 have a higher metastatic potential, though it does not mean quicker onset or initiation of the tumor [17]. We have also previously shown that MIEN1 increases phosphorylation of Akt causing the translocation of NF-κB to the nucleus and then transcriptionally activating downstream effectors like MMP-9, uPA and VEGF [15]. These proteases and angiogenic factors are known to cleave extracellular matrix, hence facilitating migratory and invasive potential of the cells. Taken together, these studies confirm that MIEN1 plays an important role in the progression of cancer rather than in the initiation of the tumor. Here, we show that upon ectopic reintroduction of miR-940, the mRNA as well as protein levels of the effector molecules decrease in DU-145. Conversely, downregulation of the miRNA using inhibitors increases the effector molecules in PWR-1E. Hence, miR-940, indirectly through MIEN1, is capable of decreasing expression levels of specific proteins that facilitate migration and invasion.

It is known that miRNAs can affect expression by causing mRNA degradation through complex formation with RNA-induced silencing complex or by repressing the translation of the mRNA, thus inhibiting formation of the protein [22]. The degree of complementarity and the competitive endogenous mRNAs determine the fate of the miRNA-mRNA complex [24]. Here, we see that miR-940 expression is highest in the immortalized PWR-1E cells, followed by PC-3 cells and lowest in DU-145. In addition to this expression pattern, our data prove that inhibition of miR-940 has different effects on MIEN1 mRNA and protein levels in the various cell lines. This implies that the inhibition of MIEN1 using miR-940 affects MIEN1 in a manner dependent on not only the cellular context (other competitive endogenous mRNA in the specific cells) but also on the endogenous miRNA levels. While in PWR-1E, the endogenous miR-940 potentially degraded MIEN1 mRNA; the overexpression of anti-miR-940 resulted in attenuation of MIEN1 mRNA degradation, thus causing an increase in both MIEN1 mRNA and protein levels. Conversely, the loss of the endogenous miR-940 in DU-145 possibly led to the overexpression of both MIEN1 mRNA and protein; and hence ectopic overexpression of the miR-940, as we have observed, caused MIEN1 mRNA degradation resulting in significant depletion of both transcript and protein levels. However, in PC-3, where MIEN1 mRNA is expressed but protein is low, inhibition of miR-940 with anti-miR-940 resulted in no further increase of MIEN1 mRNA but only increased MIEN1 protein. Additionally, the inhibition of endogenous MIEN1 mRNA with ectopic miR-940 in PC-3 decreased MIEN1 transcript. Together, this indicates that the endogenous miR-940 was causing translational repression of the MIEN1 mRNA rather than degradation in PC-3. Thus, these results show that while miR-940 causes MIEN1 mRNA degradation in DU-145 (ectopic and conceivably endogenous expression) and PWR-1E (endogenous), it causes translational repression of MIEN1 in PC-3 cells (endogenous). The unique ability of this miRNA to perform both mRNA degradation as well as translational repression of the same target depending on the levels of the miRNA and the cellular context seems like a novel finding.

MicroRNAs could target multiple transcripts, thus eliciting a response which is dependent on the combined effects on its targets [24]. A recent study implied that miR-940 could be one of the regulators of alpha-1 antitrypsin [34]. Loss of this serine proteinase inhibitor results in increased risk of lung and liver cancers while its elevated serum levels are associated with prostate cancer [35], supporting our hypothesis that the miR-940 is lost in cancer cells and tissues. In our study, we performed experiments to determine the effects miR-940 would have on migration and invasion, thus delineating the mechanism by which miR-940 could affect cancer progression, based on its regulation of MIEN1, a validated player in the regulation of prostate cancer migration and invasion. We observed a decrease in both the migratory and invasive potential of the cells upon ectopic expression of the miRNA and the converse was seen when the miRNA was inhibited. Additionally, these effects were negated upon re-introduction of the non-targetable MIEN1 into the system. Hence, we are the first to report that miR-940 inhibits prostate cancer migration and invasion, at least in part via MIEN1 along with other probable targets. The ability of cells to form disseminated colonies without attaching to the substratum is very important to determine the tumorigenicity of the cells [26, 27, 36]. Previous reports indicate that MIEN1 enhances EMT in breast cancer [33]. Our study demonstrates that miR-940 completely inhibits this ability of prostate cancer cells along with promoting MET by increasing the E-cadherin and decreasing Vimentin expression. The decrease in slug, an indicator of cells losing their mesenchymal trait [37], was also observed in corroboration. Since EMT is a very complex yet important process in prostate cancer progression [38–40], further investigation to identify the exact mechanism by which miR-940 facilitates this transition is required. It is also important to determine the different global pathways and the proteins that may be altered by miR-940 that culminates in miR-940 mediated inhibition of prostate cancer progression. Since miR-940 is a very novel miRNA whose function has never been validated or reported in any pathway before, in our study we used a set of common genes predicted to be targets of miR-940 by multiple algorithms. The extensive gene list was then categorized into known and validated pathways using the Kyoto Encyclopedia of Genes and Genomes (KEGG) pathway mapping and annotation table available in the Database for Annotation, Visualization and Integrated Discovery 6.7 (DAVID 6.7) [41, 42]. The preliminary examination of the results obtained from DAVID was then represented as a function of the number of genes involved within the pathway that could be downregulated by miR-940 (Additional file 6: Table S1A), and further classified based on the significance of the overall pathway alteration (Additional file 6: Table S1B). Interestingly, the pathway with most number of genes affected was a global pathway in cancer. Also, many other predictions within the threshold set indicated the regulation of other pathways considered important in cancer. Thus, miR-940 may be eliciting the responses we have observed via other targets in addition to MIEN1 and this needs further validation.

Studies over the past decade have proven beyond question that in addition to understanding the regulation of pathways, early stage identification and targeting of prostate cancer is of primary importance in order to prevent metastasis of the disease. Use of deregulated miRNA profiles is currently under consideration to enable the advancement of detection and diagnoses [10, 28–30]. We have seen that miR-940 expression is high in the normal and benign glands in tissues obtained from patients who have undergone prostatectomy compared to lower expression in the tumor. Associating the expression patterns of miR-940 and MIEN1 with the early detection and differentiation of the indolent from aggressive disease will be a valuable tool that could be used clinically for early prostate cancer detection. Use of circulating miRNAs from the serum is not only minimally invasive but also reliable since miRNAs are highly stable in the blood and hence can be used as potential biomarkers [43, 44]. Apart from the potential use of miR-940 in tissues and serum as a biomarker along with the expression of MIEN1, studying the regulation of miR-940 itself may provide more insight into the mechanism of its expression pattern and the reasons for its loss in cancer, which from our results, indicates facilitation of cancer progression. Examination of miR-940 promoter exhibited high possibility of methylation and this warrants further investigation.

## Conclusions

This study is the first to identify miR-940 as a novel regulator of MIEN1, a molecule involved in prostate cancer progression. With our *in vitro* studies, we established the role of miR-940 in several key processes of metastasis; including migration, invasion, anchorage-independent growth and EMT. Additionally, with the clinical investigations in a small sample cohort, we demonstrated that miR-940 expression is low in tumor, contrary to MIEN1 expression pattern. Together, this could be an important regulator-target combination to study and be used as prognostic indicators for prostate cancer.

## Materials and methods

### Cell lines, cell culture, siRNA, miRNA and plasmid transfections

Human prostate carcinoma cells DU-145 (ATCC HTB-81), PC-3 (ATCC CRL-1435), and LNCaP (ATCC CRL-1740) were maintained in RPMI 1640 media supplemented with 10% fetal bovine serum (Life Technologies). Immortalized non-tumorigenic prostate epithelial cell line HPV-18C-1 (a kind gift from Dr. Jhong S. Rhim, Frederick Cancer Research and Development Center, National Cancer Institute, Frederick, MD) and PWR-1E (ATCC CRL-11611) were maintained in Keratinocyte-SFM (Life Technologies) supplemented with bovine pituitary extract (25 μg/ml) and recombinant epidermal growth factor (0.15 ng/ml). Cells were cultured at 37°C with 5% CO_2_. The cell lines were authenticated according to “Authentication of Human Cell Lines: Standardization of STR Profiling” using *GenePrint*® 10 System (Promega); all cell lines and their passages exhibited >80% match to the initial cell line STR profile provided by ATCC [45]. The smart pool siRNAs were obtained from Dharmacon (Thermo Fisher Scientific), while the precursor and inhibitor miRNA oligos (Pre- and Anti-miR) were purchased from Ambion (Life Technologies). The final concentration of the miRNA oligos used for transfection was determined by preliminary concentration-dependent studies and remained constant for all the experiments. Plasmid transfections were performed using Lipofectamine 2000 while Lipofectamine RNAiMAX was used for RNAi transfections, performed according to the manufacturer’s protocols (Life Technologies).

### Antibodies and reagents

The following antibodies and reagents were used: Mouse monoclonal and mouse polyclonal MIEN1 (Abnova; antibody specificity tested and proven in previous studies[15, 17]), rabbit polyclonal MIEN1 (Life Technologies; antibody specificity tested in previous studies[15]), mouse monoclonal GAPDH (Santa Cruz Biotechnology), rabbit monoclonal pNF-κB p65 S536 and rabbit polyclonal MMP-9 (Cell Signaling Technology), mouse monoclonal VEGF and uPA (R&D Systems), mouse monoclonal Alexa Fluor 594 conjugated Phalloidin (Life Technologies), mouse monoclonal E-cadherin (BD Biosciences), Vimentin (supernatant developed in mouse and tested against human antigen, Developmental Studies Hybridoma Bank), anti-mouse and anti-rabbit IgG (Promega), AlexaFluor 488 goat anti-mouse IgG and AlexaFluor 594 goat anti-mouse IgG (Life Technologies) sheep anti-DIG-AP antibody and NBT-BCIP ready-to-use tablets (Roche), sheep serum (Jackson ImmunoResearch), rabbit IgG, BSA, levamisole hydrochloride, Tris-HCl (pH 7.4), nuclease free water, SSC buffer, Xylene, Tween-20, Nuclear Fast Red, Hematoxylin and Eosin (Sigma-Aldrich) and Permount and PBS (Thermo Fisher Scientific).

### Bioinformatics and microarray analysis

*In silico* analyses were performed to determine the putative miRNAs that could target MIEN1. The software programs used included miRANDA [20], PicTar [46], miRBase [47] and TargetScan [21], all of which used the 3′UTR as the target region to determine miRNA recognition elements and provided scores to determine predictive values.

For microarray based hybridization, DU-145 and PWR-1E cells were trypsinized, spun down, washed with sterile PBS and frozen immediately at -80°C. The samples were de-identified and shipped to LC Sciences (Houston, TX) for microarray hybridization. In brief, total RNA was isolated from the cells and enriched for small RNA (<300nt). Subsequently, the small RNAs were 3′ extended with polyA tail and an oligonucleotide tag was ligated to it for fluorescent dye staining (Cy3). The samples were then hybridized to the probe set on the plate (probes consisted of sequences complementary to miRNA from miRBase as well as the specially requested custom probes). After hybridization, the miRNA expression was detected by fluorescence labeling using tag-specific dye. Images collected were analyzed using Array-Pro image analysis software. Data analysis involved subtraction of the background along with normalization. Paired t-test results were provided for further interpretation and study.

### qPCR

Total RNA was isolated from the cell lines using TRIzol (Life Technologies) and quantified. Equal amount of RNA was used for the one-step or two-step qPCR performed using the Superscript III SYBR Green qRT-PCR kits, according to manufacturer’s instructions (Life Technologies). For miRNA, PCR was performed using NCode VILO miRNA cDNA Synthesis and EXPRESS SYBR GreenER miRNA qRT-PCR Kits (Life Technologies), according to the manufacturer’s protocol. The primers (sequences provided in the Supplementary materials and methods; Additional file 7) were designed using Primer 3 [48] and synthesized by Integrated DNA Technologies (Coralville, IA). PCR was performed using Realplex^2^ Mastercycler ep *gradient* S thermal cycler (Eppendorf).

### Western blotting

Western blotting was performed according to standard protocols. Briefly, total protein was isolated using NP-40 lysis buffer and estimated using the standard Micro BCA Protein Assay Kit (Pierce Biotechnology). NuPAGE® Novex® 4-12% Bis-Tris Gels were used and the samples were transferred onto nitrocellulose membranes using an iBlot (Life Technologies). Membranes were blocked in 5% non-fat dry milk or 1% BSA prior to antibody subjection. The chemiluminescent reaction was captured by the AlphaImager (ProteinSimple) and bands were analyzed using ImageJ software [49].

### Northern blotting

Northern blotting was performed using miRNA Northern Blot Assay Kit and custom ordered biotin-labeled miR-940 and U6 control probes (Signosis) with one microgram of total RNA from each cell line, according to manufacturer’s instructions.

### RNA stability assay

Cells were transfected with the precursor oligomiRs and 48 hours after transfection, treated with 10 μg/ml Act-D (Sigma-Aldrich). RNA was isolated at several time points and quantified. Equal amounts of RNA were used to run qPCR to determine MIEN1 levels.

### Luciferase reporter assay

Cells were transfected with 3′UTR luciferase constructs (Origene) - Empty Vector (Vec) or 3′UTR-MIEN1 (MIEN1^WT^ / MIEN1^Mut^) and miR-940 or miR-NT in duplicate. Luciferase assay was performed using the Luciferase Assay System (Promega) according to manufacturer’s instructions and luminescence read using Synergy2 Alpha Microplate Reader (BioTek).

### Migration assay

For migration assay, a scratch was made in a monolayer of transfected cells using a pipet tip, 48 hours after transfection. Fresh media was added immediately to remove the floating cells and the scratch and surrounding cells were imaged at T0 (immediately after scratching). Images were captured at specific time points from at least ten independent fields to determine the wound closure. Migration was calculated as a percentage of the area covered by the cells compared to the original wound area.

### Invasion assay

Invasion assay was performed with transwell invasion assay inserts and 24-well plates (BD Biosciences) according to manufacturer’s protocol. In brief, cells were transfected with the miRNA oligomiRs and the inserts were coated with Matrigel (BD Biosciences). Cells were trypsinized 48 hours after transfection and 500 μl of the cell suspension (concentration of 5 X 10^4^ cells/ml) was plated in duplicate in Matrigel-coated and non-coated transwell inserts with fetal bovine serum as a chemoattractant in the bottom well. The lower side of the transwell membranes were fixed and stained with 0.05% crystal violet 24 hours after plating. Fold change in invasion was calculated as a ratio of cells invading the Matrigel matrix-coated insert membrane to the cells migrating through the uncoated membrane. The invasion of no-targeting miRNA transfected cells was considered as 1 and the fold change was calculated accordingly.

### Flow cytometry

DU-145 cells were transfected with miRNA mimics or siRNA against MIEN1 and subjected to cell cycle analysis using Propidium Iodide in a Beckman Coulter Cytomics FC 500 Flow Cytometer. In brief, transfected cells were trypsinized, washed with PBS, counted and resuspended to a concentration of 1.5 × 10^6^cells/ml, 72 hours after transfection. Cells were fixed in cold ethanol at 4°C, overnight. After washing with PBS and centrifuging the suspension, the pellet was resuspended in PI with RNaseA and incubated at 4°C for about 3 hours in the dark before analysis.

### Anchorage-dependent and -independent growth assays

For the anchorage-dependent clonal assay, cells were treated with precursor miR-NT/940 for 48 hours and seeded (2500 cells per well) on polystyrene coated 6-well plates. After 12 days, the colonies were fixed and stained with 0.05% crystal violet or subjected to immunofluorescence. Only individual colonies (>50 cells per colony) were considered to obtain the average number of colonies for each treatment.

For anchorage-independent colony formation assay, cells were treated with precursor miR-NT/940 for 48 hours before re-plating (5000 cells per ml per well) on soft agar (cells in 2X media:agar = 1:1). Colonies were stained with 0.05% crystal violet and counted after 12 days of incubation in soft agar.

### Immunofluorescence and confocal microscopy

Cells were transfected, as described in anchorage-dependent assay, plated on coverslips to at least 90% confluence, fixed with 4% paraformaldehyde, permeabilized with 100% methanol, blocked with 1% BSA and stained for the specific proteins. The coverslips were mounted using PermaFluor Mountant (Thermo Fisher Scientific) and imaged using a Zeiss confocal microscope LSM 510 under 40X, water immersion objective. At least five independent fields per experiment were captured. The images were analyzed with LSM software and the predominant pattern is represented here.

### In situ hybridization and immunohistochemistry

Archived paraffin-embedded prostate tumor with matched normal and tumor infiltrating normal gland tissue sections from multiple patients were collected under the approval of the Institutional Review Board at the site. The study protocol was approved by the Institutional Review Board at UNT Health Science Center. The anatomic pathologists independently read the slides and graded the Hematoxylin & Eosin (H&E) stained sections to provide scores (1-5; based on predominant primary Gleason pattern) and read the hybridized sections to determine miR-940 intensity scores (1-5; 1 being basal to very low to 5 being high intensity) for the matched normal and prostate progression sections; a chromogenic assay based on DIG labeled probes detected by alkaline phosphatase conjugated anti-DIG and NBT-BCIP substrate was used for miR-940 staining. The Exiqon (Denmark) miRCURY LNA™ microRNA ISH Optimization Kit (FFPE) was used to standardize and perform *in situ* hybridization, using scrambled miRNA and the 5′- and 3′-DIG double labeled miR-940 probes. The extent of Proteinase-K treatment, the hybridization time and temperature, and incubation with the substrate were all standardized for the probes. Correspondingly, MIEN1 and isotype-specific rabbit IgG antibodies were used for immunohistochemistry that was performed on the serial sections according to standard protocols. The images were captured as described previously [50]. ImageJ analysis of the staining intensities in the various tissues was performed with the “Colour Deconvolution” plugin [49].

### Statistical analyses

The results were represented as mean ± S.E.M of independent experiments. The *p-value* was calculated according to Student’s t-test when comparing two groups using GraphPad P-value calculator. Multiple groups were compared by one-way ANOVA, when necessary, followed by pair-wise comparisons with post-hoc test. The differences were considered significant if *p-value* was at least ≤0.05.

### Consent

The patients provided consent for the use of the tissues for research and publication.

## Electronic supplementary material

Additional file 1: Figure S1: Post-transcriptional regulation of MIEN1. **(A)** Drosha, Dicer and MIEN1 expression levels upon knockdown of miRNA maturation enzymes, Drosha and/or Dicer compared to control siRNA (NT) in HEK293T as shown by qPCR. **(B)** Fold change in MIEN1 protein levels upon knockdown of miRNA maturation enzymes, Drosha and/or Dicer compared to control siRNA (NT) in PC-3. **P ≤0.01. (PDF 61 KB)

Additional file 2: Figure S2: Potential miRNA regulators of MIEN1. **(A)** miRNA identified by miRNA microarray*, in silico* algorithms and BLAST showing putative binding sites in the 3′UTR of MIEN1. **(B)** hsa-miR-940 precursor miRNA – stem-loop-stem structure. **(C)** Conservation of miR-940 between different species. (PDF 127 KB)

Additional file 3: Figure S3: miR-940 and MIEN1 expression patterns in various prostate normal and cancer tissues. **(A)** Pictorial representation of the miR-940 and MIEN1 expression obtained by *in situ* hybridization and immunohistochemical staining. **(B)** Graphical representation of the staining intensities for miR-940 **(B,i)** and MIEN1 **(B,ii)** based on ImageJ quantification of the tissues in the tissue microarray containing benign prostatic hyperplasia(BPH), matched normal(MN) to prostate tumors (PCa) and metastatic (Mets) tissues. Scale bar: 66.67μm. ***P ≤0.001. (PDF 335 KB)

Additional file 4: Figure S4: >miR-940 does not alter the cell viability. **(A-B)** MTT assay to determine % Viability in **(A)** PC-3 upon transfection of Anti-miR-940 and **(B)** DU-145 upon transfection of Pre-miR-940 or siMIEN1. **P ≤0.01. (PDF 62 KB)

Additional file 5: Figure S5: miR-940 attenuates EMT and promotes MET. **(A)** Morphology of colonies formed by Pre-miR-NT or Pre-miR-940 transfected DU-145 cells on adherent plates. **(B)** Slug mRNA expression in DU-145 cells transfected with Pre-miR-940 or Pre-miR-NT. **(C)** E-cadherin transcript levels in PC-3 cells when transfected with Anti-miR-940 or Anti-miR-NT. **P ≤0.01; *P ≤0.05. (PDF 82 KB)

Additional file 6: Table S1: Predicted pathways altered by miR-940. **(A-B)** Pathways potentially affected by common genes predicted by multiple algorithms as identified by KEGG pathways generated in DAVID based on the number of genes present in the pathway **(A)** and the P-value of the pathways with highest number of genes **(B)**. (XLSX 11 KB)

Additional File 7: **Supplementary materials and methods** [51, 52]. (PDF 152 KB)

## Abbreviations

3′UTR: 3′ untranslated region
Act-D: Actinomycin-D
ANOVA: Analysis of variance
Anti-DIG-AP: Anti-digoxigenin antibodies, conjugated to alkaline phosphatase
BLAST: Basic Local Alignment Search Tool
BPH: Benign prostatic hyperplasia
BSA: Bovine serum albumin
cDNA: complementary DNA
EMT: Epitelial-to-mesenchymal transition
FBS: Fetal bovine serum
GAPDH: Glyceraldehyde 3-phosphate dehydrogenase
GGTase-I: Geranylgeranyl transferase I
H&E: Hematoxylin and eosin
HER2/neu: Human epidermal growth factor receptor 2
IgG: Immunoglobulin G
LNA: Locked nucleic acid
MET: Mesencymal-to-epithelial transition
Mets: Metastatic tissue
MIEN1: Migration and invasion enhancer 1
miR: miRNA or MicroRNA
miR-940: hsa-miR-940
MN: Matched normal tissues
MMP-9: Matrix metallopeptidase 9
mRNA: Messenger RNA
MTT: 3-(4,5-dimethylthiazol-2-yl)-2,5-diphenyltetrazolium bromide
NBT-BCIP: Nitro-blue tetrazolium with 5-bromo-4-chloro-3′-indolyphosphate
NF-κB: Nuclear factor kappa-light-chain-enhancer of activated B cells
PBS: Phosphate-buffered saline
PCa: Prostate Cancer
qPCR: Real time polymerase chain reaction
qRT-PCR: Real-time reverse-transcription PCR
RNA: Ribonucleic acid
siRNA: Small interfering RNA
SSC: Saline-sodium citrate
U6snRNA: Small nuclear RNA component of U6 small nuclear ribonucleoprotein
uPA: urokinase-type plasminogen activator
VEGF: Vascular endothelial growth factor.
