# Supplementary figures and images for: MicroRNA-940 suppresses prostate cancer migration and invasion by regulating MIEN1
Source: Mol Cancer. 2014 Nov 19;13:250. doi: 10.1186/1476-4598-13-250 (PMC4246551; doi:10.1186/1476-4598-13-250)

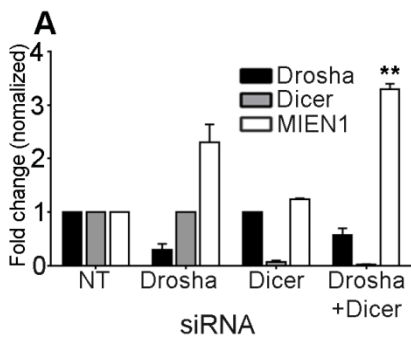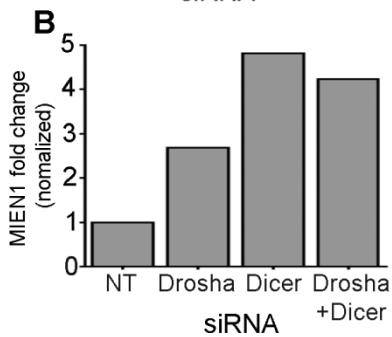

Supplement: Supplementary file 1 — Additional file 1: Figure S1: Post-transcriptional regulation of MIEN1. (A) Drosha, Dicer and MIEN1 expression levels upon knockdown of miRNA maturation enzymes, Drosha and/or Dicer compared to control siRNA (NT) in HEK293T as shown by qPCR. (B) Fold change in MIEN1 protein levels upon knockdown of miRNA maturation enzymes, Drosha and/or Dicer compared to control siRNA (NT) in PC-3. **P ≤0.01. (PDF 61 KB) [file 12943_2014_1448_MOESM1_ESM.pdf]

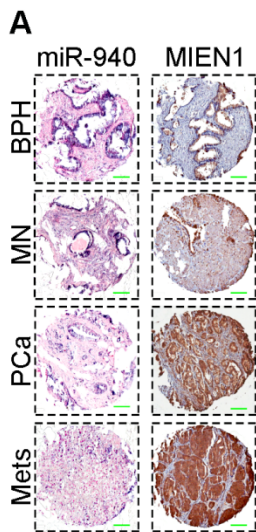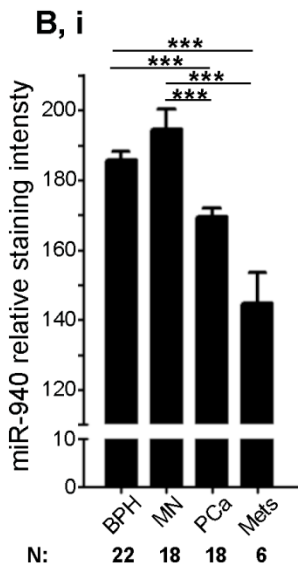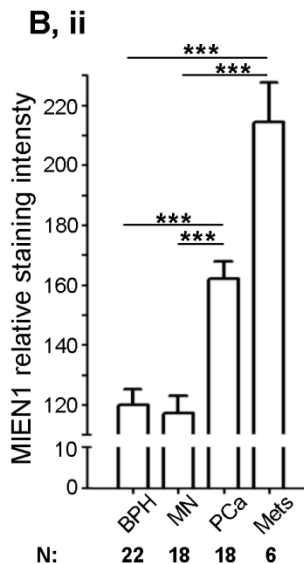

Supplement: Supplementary file 3 — Additional file 3: Figure S3: miR-940 and MIEN1 expression patterns in various prostate normal and cancer tissues. (A) Pictorial representation of the miR-940 and MIEN1 expression obtained by in situ hybridization and immunohistochemical staining. (B) Graphical representation of the staining intensities for miR-940 (B,i) and MIEN1 (B,ii) based on ImageJ quantification of the tissues in the tissue microarray containing benign prostatic hyperplasia(BPH), matched normal(MN) to prostate tumors (PCa) and metastatic (Mets) tissues. Scale bar: 66.67μm. ***P ≤0.001. (PDF 335 KB) [file 12943_2014_1448_MOESM3_ESM.pdf]

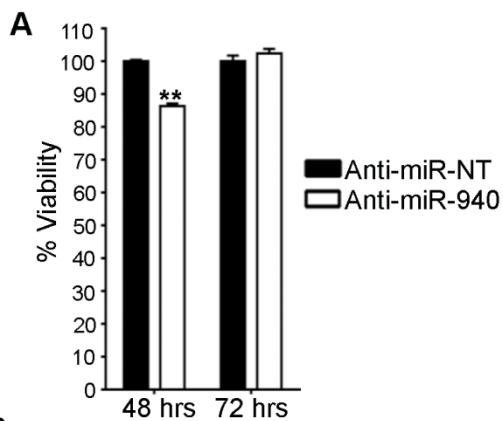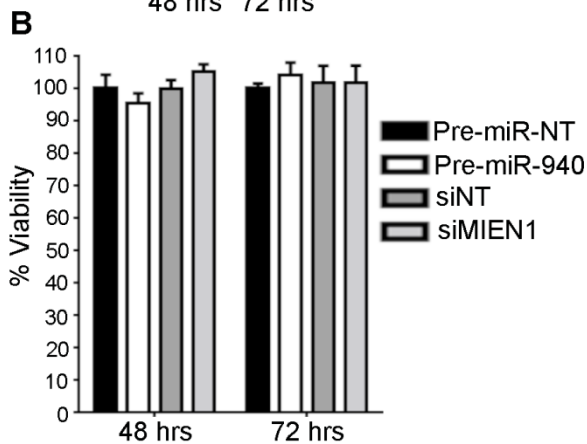

Supplement: Supplementary file 4 — Additional file 4: Figure S4: >miR-940 does not alter the cell viability. (A-B) MTT assay to determine % Viability in (A) PC-3 upon transfection of Anti-miR-940 and (B) DU-145 upon transfection of Pre-miR-940 or siMIEN1. **P ≤0.01. (PDF 62 KB) [file 12943_2014_1448_MOESM4_ESM.pdf]

**A**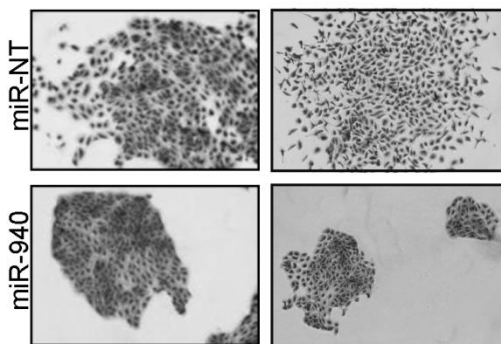**B**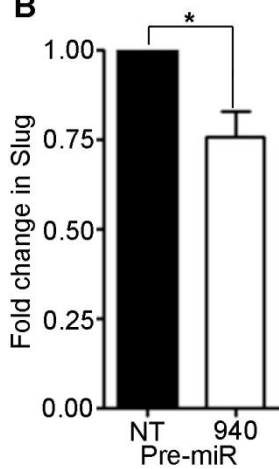**C**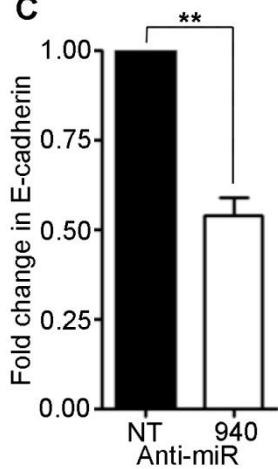

Supplement: Supplementary file 5 — Additional file 5: Figure S5: miR-940 attenuates EMT and promotes MET. (A) Morphology of colonies formed by Pre-miR-NT or Pre-miR-940 transfected DU-145 cells on adherent plates. (B) Slug mRNA expression in DU-145 cells transfected with Pre-miR-940 or Pre-miR-NT. (C) E-cadherin transcript levels in PC-3 cells when transfected with Anti-miR-940 or Anti-miR-NT. **P ≤0.01; *P ≤0.05. (PDF 82 KB) [file 12943_2014_1448_MOESM5_ESM.pdf]
