# Supplementary material for: MicroRNA-940 suppresses prostate cancer migration and invasion by regulating MIEN1
Source: Mol Cancer. 2014 Nov 19;13:250. doi: 10.1186/1476-4598-13-250 (PMC4246551; doi:10.1186/1476-4598-13-250)

A

miRNA	p - Value	Log_2 (PWR-1E DU-145)	5' ---- MIEN1 3'UTR ---- 3' / | | | | | | | / 3' miRNA 5'	Prediction Tool
hsa-miR-324-3p	4.65 e-01	0.60	GAUUCCGUGGCC UUGGGGGCAGG<br>GGUCGUCGUGGAC CCCGUCA<br>TCGAGTCGCGCC TCGGGGGCACAG<br>GGUCGUCGUGGACCCCGUCA	miRanda, TargetScan, Micro-Inspector
hsa-miR-221	9.64 e-03	0.64	AACCCAG--GGCAATGT CAGCT<br>UUGGGUCGUCUGUUACA-UCGA	miRanda
hsa-miR-940	3.63 e-02	0.90	GCUGGGAGCUCCC--C CUGCCUC<br>CCCCUCGCCCCCGGGACGGAA	TargetScan miRanda

B

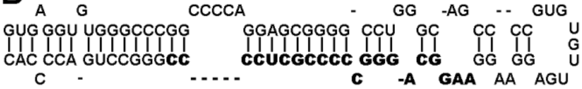

C

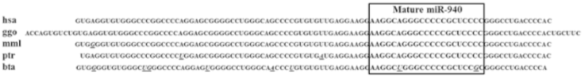

Supplement: Supplementary file 2 — Additional file 2: Figure S2: Potential miRNA regulators of MIEN1. (A) miRNA identified by miRNA microarray, in silico algorithms and BLAST showing putative binding sites in the 3′UTR of MIEN1. (B) hsa-miR-940 precursor miRNA – stem-loop-stem structure. (C) Conservation of miR-940 between different species. (PDF 127 KB) [file 12943_2014_1448_MOESM2_ESM.pdf]
