# Supplementary material for: MicroRNA-940 suppresses prostate cancer migration and invasion by regulating MIEN1
Source: Mol Cancer. 2014 Nov 19;13:250. doi: 10.1186/1476-4598-13-250 (PMC4246551; doi:10.1186/1476-4598-13-250)
Supplement: Supplementary file 7 — Additional File 7: Supplementary materials and methods [51, 52]. (PDF 152 KB) [file 12943_2014_1448_MOESM7_ESM.pdf]

Supplementary materials and methods

Primer list

MIEN1FP - 5’*CAGTGCTGTGGAGCAGT*3’,
MIEN1RP - 5’*GACGGCTGTTGGTGATCTTT*3’;
GAPDHFP - 5’*GAGCGAGATCCCTCCAA*3’,
GAPDHRP - 5’*ACTGTGGTCATGAGTCCTTC*3’;
MMP-9FP - 5’*TTGACAGCGACAAGAAGTGG*3’,
MMP-9RP - 5’*GCCATTCACGTCGTCCTTAT*3’;
uPAFP - 5’*TGCGTCCTGGTCGTGAGCGA*3’,
uPARP - 5’*CTACAGCGCTGACACGCTTG*3’;
VEGFFP - 5’*CCTGGTGGACATCTTCCAGGAGTA*3’,
VEGFRP - 5’*CTCACCGCCTCGGCTTGTCACA*3’;
E-cadherinFP - 5’*CGGGAATGCAGTTGAGGATC*3’,
E-cadherinRP - 5’*AGGATGGTGTAAGCGATGGC*3’;
SlugFP - 5’*AATATGTGAGCCTGGGCG*3’,
SlugRP - 5’*CTCTGTTGCAGTGAGGGCAAG*3’

MTT assay - DU-145 and PC-3 cells were transfected with precursor oligomiRs or MIEN1 specific siRNA and inhibitor oligomiRs respectively. The cells were trysinized, counted and plated in complete media on a 96-well plate, 24 hours after transfection. Percent viability was measured 48 and 72 hours after reseeding by 3-[4,5-dimethylthiazol-2yl]-2,5-diphenyltetrazolium bromide (MTT) assay. The absorbance was measured by Synergy2 plate reader (BioTek) at 570 nm wavelength.

Bioinformatic Analysis - For identification of pathways that could be affected by miR-940, a common list of genes that were predicted by four independent algorithms, miRanda [20], TargetScan [21], DIANAmT [51] and miRWalk [52], was generated. This list was then analyzed using Database for Annotation, Visualization and Integrated Discovery 6.7 (DAVID 6.7) to classify genes according to the pathways they were involved in based on the Kyoto Encyclopedia of Genes and Genomes (KEGG) pathway mapping function [41, 42]. Pathways containing 20 or more genes from the common list were first tabulated. This list was then rearranged to obtain a hierarchy of pathways with significant Fisher Exact P-values (< 0.05).
